# Tramadol-Induced Mood Elevation in a Patient with No Previous Psychiatric History

**DOI:** 10.1155/2018/9574395

**Published:** 2018-10-01

**Authors:** Mugtaba Osman, Mashael Mustafa

**Affiliations:** ^1^Armed Forces Centre for Psychiatric Care, Armed Forces Hospital in Taif Region, Taif, Saudi Arabia; ^2^Armed Forces Centre for Health Rehabilitation in Taif, Armed Forces Hospital in Taif Region, Taif, Saudi Arabia

## Abstract

Tramadol is a powerful analgesic medication with antidepressant effects like venlafaxine. Hypomanic features were reported in patients with psychiatric history of mood disturbance when tramadol was prescribed for them. However, it is extremely rare to notice such mood-elevating effect in patients who have no previous psychiatric history. We report on the observation of a distressing mood-elevating effect for tramadol in a patient with no previous psychiatric history. We present the case of a 26-year-old female patient who developed accelerated flow of speech, overactivity, and difficulty in sleeping following intake of tramadol 50 mg dose. These symptoms resolved four hours later and recurred as she retook tramadol. The patient has no history of mood disorder or any other psychiatric comorbidity. Clinicians should exercise caution when prescribing tramadol.

## 1. Introduction

Tramadol is a well-known powerful centrally acting analgesic medication with established pharmacological effects on opioid systems (mainly *μ*-opioid receptors agonism) and nonopioid systems (5-Hydroxy Tryptamine and Noradrenaline reuptake inhibition) [1]. Tramadol is, in fact, closely related to chemical structure of all dual action antidepressants, most notably venlafaxine. Thus, tramadol and venlafaxine shared monoaminergic action leading to the tramadol effects on mood [2]. Reports of antidepressant effects for tramadol have trickled in the clinical literature either in combination with other antidepressants [3, 4] or on its own [5–7] in patients with [8] and without need for analgesia [9]. There is currently growing theoretical and clinical concern in terms of drug-drug interaction when tramadol is used concomitantly with serotoninergic antidepressants [10–13].

A recent review has clearly concluded that “caution is required” if tramadol is to be prescribed for patients on antidepressants (and vice versa) with clear contraindication for the use of tramadol in patients taking Mono-Amine Oxidase Inhibitors [14]. Of note, tramadol, owing to its powerful blockade of serotonin reuptake, was reported to cause serotonin syndrome even in the absence of concomitant use of antidepressants [15, 16].

Hypomanic features were reported in patients with psychiatric history of mood disturbance when tramadol was prescribed for them [12, 17–21]. However, it is extremely rare to notice such mood-elevating effect in patients who have no previous psychiatric history [22]. In this report we describe a case of a nonpsychiatric patient who experienced distressing mood symptoms as they start taking tramadol to control her pain.

## 2. Case Presentation

We present the case of a 26-year-old female patient who developed severe persistent lower back pain secondary to spondylolisthesis in the fifth lumbar vertebra. Tramadol 50 mg dose was prescribed as a nonsurgical measure to achieve satisfactory analgesia. She took one 50 mg tablet and the pain was effectively controlled. However, she almost immediately noticed accelerated flow of speech and was unable to control her desire to talk incessantly. She was also quite overactive and “on the go” for the following four hours following intake of tramadol 50 mg dose. She noticed that she did not want to “sit still” and continued to walk to-and-fro and engaged in cleaning the house as she experienced increased energy. She was unable to sleep, although she experienced increased irritability with neither extreme happiness nor euphoria. She retook tramadol 50 mg twice 4 days and 7 days later and the same condition (overtalkativeness, overactivity, and distress) reoccurred upon both occasions lasting for exactly 4 hours each time. There was no ataxia, tremors, blurring of vision, or any other neurological signs or symptoms. She did not want to take tramadol anymore.

Notably, she had no previous psychiatric or neurological history of note. She took no psychotropic medications for any physical or psychological reasons. She had no history of illicit substance misuse or dependence.

Apart from spondylolisthesis, she suffered from urticaria and congenital optic disc tilt. She took Chlorzoxazone one tablet on request, ranitidine 150mg daily, and Desloratadine 10mg daily.

## 3. Discussion

A number of cases of hypomania and mania secondary to tramadol were published over the last few years [12, 17–22]. For instance, Sharma (*2016*) [17] described a case of female patient who developed hypomanic symptoms after taking tramadol for treatment of fibromyalgia; of note the patient had an established diagnosis of bipolar affective disorder.

Occurrence of mood symptoms after tramadol prescribing constitutes genuine concern to clinicians in terms of prediction of who would get such symptoms and how to initiate effective management for them. One recent study found that prescription of antidepressants has increased following initiation of tramadol therapy [23]. There are no studies, to the best of our knowledge, evaluating prescription of other psychotropic agents, specifically mood stabilizers, in patients who were prescribed tramadol.

What is unique in our case report is the occurrence of hypomanic-like symptoms in a patient with no history of mood disorder or any other psychiatric comorbidity. The overactivity and talkativeness in our patient were almost immediate after the intake of tramadol and they resolved within few hours afterwards. This substantially increases the likelihood of our assertion, where such hypomania-like symptoms were caused by tramadol. All other potential explanations (such as primary subthreshold mood disorder, medical condition-related mood symptoms) were extremely less likely given the history of our patient.

Mood-elevation secondary to tramadol treatment could be regarded as unwanted extension of its antidepressant effect. Many neurological pathways were proposed to have some involvement in the antidepressant effect for tramadol. In addition to serotonergic and noradrenergic receptors, tramadol could exert an antidepressant effect by its ability to bind to D2 and D3 dopaminergic receptors [24, 25] and imidazoline I2 receptors [24, 26]. Many other mechanisms implicated in the antidepressant effects of tramadol were investigated. In a recent experiment Jesse et al. [27] found evidence to suggest that the signalling pathway that encompass L-arginine-nitric oxide-cyclic guanosine monophosphate is involved in the antidepressant action of tramadol. An earlier experiment found that tramadol caused substantial reduction in the alpha-2 adrenergic receptors in all brain areas, like the effects of mirtazapine and mianserin [28]. Also, other experiments indicated that tramadol causes the alpha-1 adrenergic receptors to increase in density in the brain cortex and results in upregulation of dopaminergic receptors in the nucleus accumbens which has important functions in motivational behaviour [29].

Clinical trials' results indicate that 7% of patients taking tramadol would experience delirium-like Central Nervous System stimulation with a wide range of physical, neurological, and emotional presentations [30]. Emotional presentations recorded are mostly symptoms of hyperactive delirium and include anxiety, sleep difficulties, emotional lability, euphoria, and confusion [31, 32]. However, the precise mechanism of development of such unwanted idiosyncratic adverse reaction to tramadol remains largely unknown. Also, the proportion of patients with no previous psychiatric history who developed such emotional effects, although believed to be quite small, was unclear.

We report on the observation of a distressing mood-elevating effect for tramadol in a patient with no previous psychiatric history. This will, of course, make it prudent for clinicians to exercise caution when prescribing tramadol. Also, we recommend close follow-up after initiation of tramadol treatment, in order to screen for mood symptoms in this cohort of patients.

We also recommend that longitudinal studies be carried out to robustly evaluate the prevalence and predictors of occurrence of mood symptoms in patients using tramadol.
